# Analysis of Epidemiology and Risk Factors of Atopic Dermatitis in Korean Children and Adolescents from the 2010 Korean National Health and Nutrition Examination Survey

**DOI:** 10.1155/2017/5142754

**Published:** 2017-08-22

**Authors:** Kyung Suk Lee, In-Hwan Oh, Sun Hee Choi, Yeong-Ho Rha

**Affiliations:** ^1^Department of Pediatrics, CHA Bundang Medical Center, CHA University School of Medicine, Seongnam, Republic of Korea; ^2^Department of Preventive Medicine, College of Medicine, Kyung Hee University, Seoul, Republic of Korea; ^3^Department of Pediatrics, School of Medicine, Kyung Hee University, Seoul, Republic of Korea

## Abstract

**Background:**

Atopic dermatitis (AD) is a common chronic inflammatory skin disease, but only few studies involved samples of children and adolescents that are representative of the entire Korean population. This study aimed to estimate the prevalence and risk factors of AD among children and adolescents in Korea by using nationally representative data.

**Methods:**

We used data from the fifth Korean National Health and Nutrition Examination Survey in 2010 and retrospectively evaluated 2,116 children and adolescents. Logistic regression analysis was performed to determine the relationship between AD and other variables, including IgE levels.

**Results:**

The prevalence rate of AD in children and adolescents was 15.0%. In the multivariate analysis of ages from 1 to 18 years, age (adjusted odds ratio [aOR], 0.96; *p* < 0.01) was related to AD. From age of 12 to 18 years, smoking (aOR, 8.99; *p* < 0.01) and elevated total IgE serum level (aOR, 5.31; *p* < 0.01) were related to AD.

**Conclusion:**

Age, smoking, and elevated total IgE level were related to AD in the children and adolescents. Thus, an antismoking policy and public education are necessary for reducing the prevalence of allergic diseases. In addition, measurement of total IgE level and age may be helpful in the diagnosis of AD.

## 1. Introduction

Atopic dermatitis (AD) is the most common chronic skin disorder in children and is characterized by pruritus and eruption [1, 2]. AD is classified into extrinsic AD, which exhibits elevated total IgE (tIgE) levels in the presence of IgE-mediated sensitization to specific allergic antigens, and intrinsic AD, which exhibits normal tIgE levels in the absence of IgE-mediated sensitization to allergic antigens [3]. The typical symptom of AD is pruritus, which may cause sleep disturbance, anxiety, anger, or depressive symptoms [1, 4]. In addition, AD negatively affects patients' social life, lowers their academic achievement, and sometimes causes patients to think about committing suicide [4]. According to the International Study of Asthma and Allergies in Childhood (ISAAC), the prevalence of AD varies greatly from 0.3% to 20.5% [5]. The 2010 ISAAC in South Korea also showed that the prevalence of “AD diagnosis, ever” was 35.6% in children aged 6-7 years and 24.2% in adolescents aged 12-13 years [6].

Although there have been domestic studies on the epidemiology and risk factors of AD which have been designed to represent the entire population of South Korea, domestic studies that analyzed tIgE and specific IgE (sIgE) levels as variables are scarce [7]. Therefore, this study aimed to investigate the prevalence and risk factors of AD and to determine the association of tIgE and sIgE levels with the occurrence of AD based on data from the first year of the fifth Korean National Health and Nutrition Examination Survey (KNHANES), 2010.

## 2. Materials and Methods

### 2.1. Subjects

The fifth KNHANES is a cross-sectional survey conducted throughout the year for all household member samples aged ≥1 year from 3,840 households by sampling 192 enumeration districts each year [8]. The fifth KNHANES was performed with a rolling sampling design, including a complex, stratified, multistage probability cluster survey and was stratified into two phases [7, 8]. The survey procedure of SPSS was used considering complex sampling design of KHANES [8]. All the participants signed informed consent forms. The subjects of this study were 2,116 individuals aged 1–18 years who participated in health interview and examination surveys among 8,958 participants of the fifth KNHANES [8]. The survey was approved by the institutional review board of the Korea Centers for Disease Control and Prevention (2010-02CON-21-C) [8]. Detailed information about KNHANES is available on http://knhanes.cdc.go.kr/.

### 2.2. Variables

AD was defined as responding “yes” to the question, “Have you ever been diagnosed with AD by a physician?” Smokers were defined as those who smoked at least once within a month, and drinkers were defined as those who drank at least once within a month. Obesity was defined as a body mass index (BMI) of ≥25 kg/m^2^ or higher than the 95th percentile of sex- and age-specific BMIs in the 2007 Korea National Growth Chart for BMI (kg/m^2^) [9]. tIgE and sIgE levels were tested by using ImmunoCAP 100 (Phadia, Sweden). tIgE level was analyzed by performing log 10 transformation. Specific antigens were of 3 kinds:* Dermatophagoides farinae*, dog, and cockroach. Antigen-specific IgE levels of ≥0.35 kU/L were considered positive, and a positive result was considered indicative of sIgE sensitization [1]. Age, sex, residence, and other factors were also surveyed.

### 2.3. Statistical Analyses

Categorical variables are presented as proportions (percentages), and the chi-square test was performed for the cross-tabulation analysis of AD by using the categorical variables. Continuous variables are presented as means (±standard error), and Student's *t*-test was performed to analyze the differences of AD in terms of the continuous variables. To examine the association between AD and its risk factors, odds ratio (OR) was determined by using multiple logistic regression analysis. Primarily, all the children and adolescent subjects aged 1–18 years were analyzed. Secondarily, those aged 12–18 years were analyzed with a health behavior questionnaire about smoking and drinking status and for serum vitamin D concentrations, tIgE concentration, and sIgE sensitization. All statistical analyses were performed by using IBM SPSS ver. 21.0 (IBM Co., Armonk, NY, USA) to evaluate the stratified cluster sampling design survey. All presented results are weighted values. A *p* value of <0.05 was considered statistically significant.

## 3. Results

### 3.1. General Characteristics

**Figure 1:**
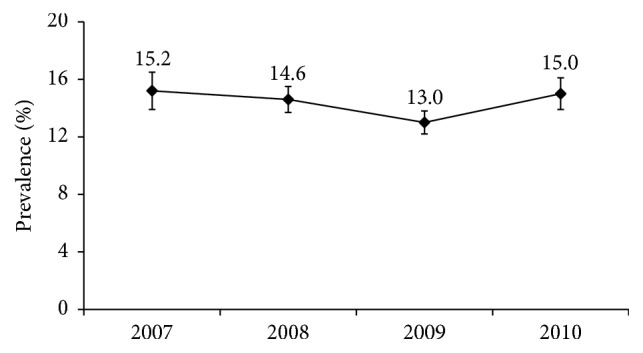
Change in prevalence of AD in children and adolescents (2007–2010). The prevalence of AD in children and adolescents was 15.2% ± 1.3% in 2007, 14.6% ± 0.9% in 2008, 13.0% ± 0.8% in 2009, and 15.0% ± 1.1% in 2010. (mean ± standard error (SE)).

The prevalence of AD in Korean children and adolescents decreased from 15.2% ± 1.3% in 2007 to 13.0% ± 0.8% in 2009 and remained unchanged at 15.0% ± 1.1% in 2010 (Figure 1 and Table 1). When analyzing the data of all the participants aged 1–18 years, the mean age of those with AD was 9.7 ± 0.4 years, while the mean age of those without AD was 10.5 ± 0.2 years, showing a statistically significantly higher mean age for those without AD (*p* = 0.02). No significant difference in the prevalence of AD was found according to sex, region, and obesity. Among the children and adolescents aged 12–18 years, the prevalence of AD was 15.2% ± 4.7% in the smokers and 11.7% ± 1.9% in the nonsmokers and was 11.4% ± 2.9% in the drinkers and 12.2% ± 2.0% in the nondrinkers. The mean serum vitamin D level was 15.6 ± 0.5 ng/ml in the patients with AD and 16.3 ± 0.4 ng/ml in the subjects without AD, showing no statistically significant difference. Twenty subjects with extrinsic AD had positive sIgE results, and 16 subjects with intrinsic AD had all-negative sIgE results. The prevalence of AD was 14.1% ± 3.8% in the subjects with positive sIgE results and 14.4% ± 3.7% in the subjects with negative sIgE results, showing no significant difference. No significant differences were found in the prevalence of AD according to the presence or absence of* D. farinae-*, dog-, and cockroach-specific sensitization. The tIgE geometric mean was 200.22 ± 1.28 kU/L in the patients with AD and 94.61 ± 1.11 kU/L in the subjects without AD, showing a statistically significant difference (*p* < 0.01; Table 1).

### 3.2. Risk Factors of AD in the Subjects Aged 1–18 Years

Among the various variables, increasing age was found to be significant (crude OR, 0.97; 95% confidence interval [CI], 0.94–0.996; *p* = 0.03). The results of the multivariate logistic regression analysis also showed that the prevalence of AD statistically significantly decreased with increasing age (adjusted OR, 0.96; 95% CI, 0.93–0.99; *p* < 0.01) (Table 2).

### 3.3. Risk Factors of AD in the Subjects Aged 12–18 Years

Smoking and drinking status, 25-hydroxyvitamin D (vitamin D) levels, tIgE levels, and sIgE sensitization were added to the variables for the subjects aged 12–18 years (Table 3). The results of the univariate analysis showed that smoking status was not significantly associated with AD (crude OR, 1.36; 95% CI, 0.62–2.96; *p* = 0.44), whereas the results of the multiple logistic analysis showed that smoking status was statistically significantly associated with AD (adjusted OR, 8.99; 95% CI, 2.03–39.79; *p* < 0.01). An increase in tIgE level was statistically significantly associated with AD in both the univariate analysis (crude OR, 2.65; 95% CI, 1.28–5.46; *p* < 0.01) and multiple logistic analysis (adjusted OR, 5.31; 95% CI, 1.81–15.61; *p* < 0.01). No significant association was found between the other variables and AD.

## 4. Discussion

This study investigated the prevalence of AD and its risk factors among Korean children and adolescents by using data from the KNHANES. We demonstrated that age, hazard behavior such as smoking, and elevated tIgE level are associated with AD in Korean children and adolescents. To our knowledge, this study is the first to analyze AD in children and adolescents by using tIgE and sIgE levels, which were measured only in the 2010 KNHANES.

In the previous epidemiological studies in South Korea, the prevalence of “itchy eczema, ever” in children aged 6-7 years increased over the past 10 years from 17.1% in 2000 to 27.0% in 2010, and the prevalence of “itchy eczema, ever” in children aged 12-13 years increased from 13.4% in 2000 to 20.6% in 2010 but tended to be low in those who were older [1, 6]. According to the Korea Youth Risk Behavior Web-based Survey, the lifetime prevalence of AD was 17.3% in 2007, 18.5% in 2008, 18.9% in 2009, and 23.1% in 2010 [9, 10]. The prevalence of AD was found to be lower than that found in the Korea Youth Risk Behavior Web-based Survey. This may be because the KNHANES minimized statistical bias compared with other researches and was conducted through a one-on-one interview with the subjects rather than a simple questionnaire or online self-administered survey, by which the respondents were possibly more cautious about their answers.

In the present study, we analyzed the association between tIgE levels and presence or absence of sIgE and allergic diseases in Korean children and adolescents. IgE has been revealed to be produced through isotype switching in B cells by IL-4 and IL-13, which are secreted from Th2 cells, and IgE production is further increased by IL-5 and IL-9 [11, 12]. Elevated IgE levels cause immune responses that interact with mast cells to express allergic diseases [13]. Therefore, elevated IgE levels can be a risk factor of allergic diseases [14]. Various studies, including the NHANES in the United States (US NHANES), demonstrated that the tIgE levels in the patients with allergic diseases were higher than those in healthy controls [15–17]. The results of this study suggest that an increase in tIgE level was associated with AD in children aged ≥12 years. However, no significant difference in prevalence was found between sIgE-positive extrinsic AD and sIgE-negative intrinsic AD. A possible explanation for such a result may be that the number of tested sIgEs was few in this study. In the US NHANES, the prevalence rates of positive IgEs were 18.5% for* D. farinae*, 10.3% for cockroach, and 11.8% for dog dander, and all possible allergens of allergic diseases were not represented [17]. Therefore, we cannot assert that the cases of negative sIgEs must be all intrinsic AD. In the US NHANES, 15 sIgEs were examined; thus, the reliability was high [17]. Therefore, if we increase the number of sIgEs in future studies, clearer results can be expected.

In the present study, we found that AD was closely associated with history of smoking (adjusted OR, 8.99; 95% CI, 2.03–39.79; *p* < 0.01). It was already reported in 1994 that mitogen-induced IL-4 production was rapidly increased in smokers [18]. Smoking increases the number of CD4 cells selectively, thereby increasing not only total leucocyte count but also CD4/CD8 ratio [19]. An increase in Th2 cells leads to increased IL-4 production and leads to increased response to environmental antigens such as pollen, animal dander, and house dust mites and to the development of major symptoms of allergic diseases [18]. The previous results support the result of the present study that identified smoking as a risk factor of AD. A previous study reported that the OR for developing AD in infants exposed to tobacco smoke in late pregnancy was 0.8 (95% CI, 0.7–0.9), indicating that the risk of developing AD was slightly decreased [20]. However, other studies reported that exposure to tobacco smoke in fetal life increased the risk of AD [21, 22]. In addition, a study with children reported that secondhand smoke from family members, including mothers, was a risk factor of AD in children [23]. The results of the present study revealed that smoking is an important risk factor of AD, suggesting the importance of smoking cessation in adolescent health care to reduce and prevent the development of AD. To prevent the development of AD, continuous smoking cessation education for middle and high school students is also considered necessary.

We found that vitamin D was not associated with AD. Vitamin D deficiency has been found to be a risk factor of allergic diseases in many studies [24–26]. Vitamin D was known to have a positive effect on the permeability barrier in the epidermis [25]. However, the role of vitamin D in allergic diseases is still controversial, as it has been reported that vitamin D intake during infancy increased the development of AD [27]. The KNHANES was a cross-sectional study measured at a single time point, although tests for serum vitamin D levels were conducted throughout the year. Thus, its interpretation has limitations. Further studies on the relationship between vitamin D and allergic diseases are considered necessary in the future.

The present study has several limitations. First, although the survey was conducted as a cross-sectional study throughout the year, the association between AD and other variables is difficult to clearly determine. Second, blood tests were not performed for children. As sIgE tests were performed only for 3 allergens and skin prick tests were not performed, the interpretation of the allergen test results has some limitation. Therefore, if allergen tests using additional and diverse allergens were performed, more reliable analyses would have been possible. Third, the number of participants was limited in terms of representing the entire Korean population of children and adolescents. However, because the KNHANES was designed to represent the whole Korean population and trained researchers obtained information through interviews, it sufficiently provided reliable data.

In conclusion, the prevalence of AD in children and adolescents is higher with younger age, which is a risk factor of the development of AD in Korean adolescents besides smoking and increased tIgE level. Therefore, early detection and management of AD are needed and more emphasis should be given to related education and prevention to reduce hazard behaviors such as smoking. Allergic serum tests such as IgE tests should be performed actively if necessary. Large-scale studies are also needed to identify the causal relationship between AD-related risk factors and serological factors in the future.

## Figures and Tables

**Table 1:** The demographic characteristics of atopic dermatitis in children and adolescents (1–18 years old).

Variable	Number	AD^†^ (SE)	non-AD (SE)	Prevalence, % (SE)	*p* value
Total	2116	322	1794	15.0 (1.1)	
AGE (year)		9.7 (0.4)	10.5 (0.2)		0.02^*∗*^
Sex					0.88
Male	1102	174	928	14.5 (1.3)	
Female	1014	148	866	15.5 (1.7)	
Region					0.65
City	1783	276	1507	15.5 (1.3)	
County	333	46	287	12.6 (2.4)	
Obesity					0.39
(−)	1785	274	1511	15.1 (1.1)	
(+)	195	36	159	16.0 (3.1)	
Smoking (≥12 years)					0.13
(−)	675	78	597	11.7 (1.9)	
(+)	52	9	43	15.2 (4.7)	
Drinking (≥12 years)					0.47
(−)	559	69	490	12.2 (2.0)	
(+)	170	18	152	11.4 (2.9)	
Vitamin D^‡^ (≥12 years, ng/ml)	651	15.6 (0.5)	16.3 (0.4)		0.21
Specific IgE sensitization (≥12 years)					0.94
(−)	126	16	110	14.1 (3.8)	
(+)	151	20	131	14.4 (3.7)	
Df IgE sensitization (≥12 years)					0.61
(−)	144	19	125	15.5 (4.3)	
(+)	133	17	116	12.9 (3.5)	
Dog IgE sensitization (≥12 years)					0.38
(−)	251	30	221	13.7 (3.2)	
(+)	26	6	20	19.3 (8.5)	
Cockroach (≥12 years)					0.49
(−)	225	30	195	13.2 (2.7)	
(+)	52	6	46	18.6 (7.5)	
Total IgE (kU/L) (≥12 years)	277	200.22 (1.28)	94.61 (1.11)		< 0.01^*∗*^

^*∗*^ *p* < 0.05. ^†^AD: atopic dermatitis. ^‡^25(OH) D.

**Table 2:** Logistic regression analysis of atopic dermatitis in children and adolescents (1–18 years old) (*n* = 1,947).

Variable	Crude OR	95% CI	*p *value	Adjusted OR^†^	95% CI	*p *value
AGE (year)	0.97	0.94, 0.996	0.03^*∗*^	0.96	0.93, 0.99	<0.01^*∗*^
Obesity						
(−)	1			1		
(+)	1.07	0.70, 1.64	0.76	1.15	0.76, 1.74	0.51

^*∗*^ *p* < 0.05. ^†^Adjusted by sex, residence, and income.

**Table 3:** Logistic regression of atopic dermatitis (AD) in adolescents (12–18 years old) (*n* = 269).

Variable	Crude OR	95% CI	*p* value	Adjusted OR^†^	95% CI	*p* value
Obesity						
(−)	1			1		
(+)	1.07	0.70, 1.64	0.76	2.00	0.59, 6.79	0.26
Smoking						
(−)	1			1		
(+)	1.36	0.62, 2.96	0.44	8.99	2.03, 39.79	<0.01^*∗*^
Specific IgE						
(−)	1			1		
(+)	1.03	0.51, 2.08	0.94	0.39	0.13, 1.19	0.10
Vitamin D^‡^	0.97	0.92, 1.02	0.22	0.93	0.85, 1.01	0.08
Total IgE^§^	2.65	1.28, 5.46	<0.01^*∗*^	5.31	1.81, 15.61	<0.01^*∗*^

^*∗*^ *p* < 0.05. ^†^Adjusted by age, sex, residence, income, and drinking. ^‡^25(OH) D. ^§^log_10_⁡transformed.
